# The development and validation of a nomogram for predicting brain metastases after chemotherapy and radiotherapy in male small cell lung cancer patients with stage III

**DOI:** 10.18632/aging.204865

**Published:** 2023-07-11

**Authors:** Baihua Yang, Wei Zhang, Jianjian Qiu, Yilin Yu, Jiancheng Li, Buhong Zheng

**Affiliations:** 1 Department of Radiation Oncology, Clinical Oncology School of Fujian Medical University, Fujian Cancer Hospital, Fuzhou 350014, China

**Keywords:** brain metastasis, small cell lung cancer, predicting model, individualized treatment, chemotherapy and radiotherapy

## Abstract

Objective: The purpose of this research was to develop a model for brain metastasis (BM) in limited-stage small cell lung cancer (LS-SCLC) patients and to help in the early identification of high-risk patients and the selection of individualized therapies.

Methods: Univariate and multivariate logic regression was applied to identify the independent risk factors of BM. A receiver operating curve (ROC) and nomogram for predicting the incidence of BM were then conducted based on the independent risk factors. The decision curve analysis (DCA) was performed to assess the clinical benefit of prediction model.

Results: Univariate regression analysis showed that the CCRT, RT dose, PNI, LLR, and dNLR were the significant factors for the incidence of BM. Multivariate analysis showed that CCRT, RT dose, and PNI were independent risk factors of BM and were included in the nomogram model. The ROC curves revealed the area under the ROC (AUC) of the model was 0.764 (95% CI, 0.658-0.869), which was much higher than individual variable alone. The calibration curve revealed favorable consistency between the observed probability and predicted probability for BM in LS-SCLC patients. Finally, the DCA demonstrated that the nomogram provides a satisfactory positive net benefit across the majority of threshold probabilities.

Conclusions: In general, we established and verified a nomogram model that combines clinical variables and nutritional index characteristics to predict the incidence of BM in male SCLC patients with stage III. Since the model has high reliability and clinical applicability, it can provide clinicians with theoretical guidance and treatment strategy making.

## INTRODUCTION

Lung cancer is a major global health problem and remains the leading cause of cancer-related mortality worldwide [1]. Most cases of lung cancer fall into one of two categories: either non-small cell lung cancer (NSCLC), which makes up 85 percent of all lung cancers, or small cell lung cancer (SCLC), which accounts for 15 percent [2]. Approximately 35,000 new cases of SCLC are identified each year, with the vast majority of patients being male [1]. Nearly 40% of all cases of SCLC are classified as limited-stage SCLC (LS-SCLC). SCLC is distinguished by its rapid progression and early onset of widespread metastases, both of which are associated with poor clinical outcomes. In this population, the disease’s estimated 5-year overall survival rate is less than 10% [3]. Those diagnosed with SCLC have a median survival time of 16–24 months [4]. Few advancements have been made in recent decades to enhance the survival rate of SCLC patients. Multimodal treatments, including surgery, chemotherapy, and radiotherapy, remains the standard management for treating this disease. Despite an early responsiveness to chemotherapy, most patients quickly develop therapy resistance, which leads to relapse and ultimately death [5]. Moreover, the surgical role in the treatment of SCLC is currently regarded as being very limited.

At the time of initial diagnosis, approximately 30% of patients with small cell lung cancer have no evidence of metastasis [6]. High rates of lung cancer mortality are often attributable to distant metastases, which occur in the vast majority of patients with SCLC [7]. Metastasis from SCLC is common in many organs, but the brain, bone, adrenals, liver, and lungs are particularly vulnerable targets [8]. For patients with SCLC, the brain is a favorable site for the disease to fail [9]. The possible reason is that most chemotherapeutic drugs are unable to reach the brain because of the blood-brain barrier [10]. Within the first two years after being diagnosed with SCLC, up to fifty percent of patients are at risk of developing brain metastases (BM) [11]. Due to SCLC’s radiosensitivity, whole brain radiotherapy has become the treatment of choice for patients with brain metastases [12]. For such patients, in addition to whole brain radiotherapy and conventional chemotherapy, there are various treatment methods, but the curative effect of these options is not ideal. In patients with brain metastases, early detection and intervention could improve clinical outcomes. Therefore, early identification of risk factors for brain metastasis in SCLC is crucial. The nomogram model integrates critical clinical and pathological features of the tumor and presents them in an intuitive graphical format, giving patients a personalized evidence-based risk assessment and assisting clinicians in choosing the best treatments. As a result, it is regarded as a trustworthy tool for visualizing and evaluating risk. To make metastatic screening easier, there is an urgent need for a predictive nomogram based on the clinicopathologic characteristics of SCLC patients.

To the best of our knowledge, there are few studies on the risk factors for brain metastasis in LS-SCLC. It is still unclear how to distinguish between patients’ risks of developing brain metastasis in LS-SCLC. LS-SCLC patients typically present in stage III [13]. Since most SCLC patients are male and at stage III, we present the first study to examine the risk factors for brain metastasis in male SCLC patients with stage III. There is no relevant prediction model has particularly been constructed to predict BM for this subgroup population till date. It represents the first attempt to develop prediction model for this specific population segment. The purpose of this research was to develop a predicted model for brain metastasis in male SCLC patients with stage III and helped in the early identification of high-risk patients and the selection of individualized therapies. This study offers theoretical direction for clinical individualization treatment in LS-SCLC.

## RESULTS

### Baseline characteristics of the study cohort

Overall, 112 male SCLC patients at stage III treated with chemotherapy and radiotherapy were enrolled in our study. The clinicopathologic characteristics, demographics, and therapeutic information were shown in Table 1. Patients’ age, tumor location, smoke, concurrent chemoradiotherapy (CCRT), cycle of chemotherapy before radiotherapy, total cycle of chemotherapy, RT dose, radiotherapy time, PNI, PAR, PLR, NLR, LLR, dNLR, SII, and SIRI were collected. The optimal cut-off value for age, radiotherapy time, PNI, PAR, PLR, NLR, LLR, dNLR, SII, and SIRI was calculated to be 66, 26, 45.15, 4.03, 162.81, 2.2, 4.32, 1.69, 815.1, and 0.27, respectively. The median and average age at diagnosis was 60 years and 59 years, respectively. The majority of patients (83%) less than 66 years. Of 112 patients in the study, 61.6% had a history of smoke. During the follow-up period, of 112 patients treated with CRT, twenty-eight (25%) patients developed BM.

**Table 1:** Patient demographics and clinical characteristics.

**Clinicopathologic variable**	**Total (*N*)**	**Percentage (%)**
Age (years)		
<66	93	83.0%
≥66	19	17.0%
Tumor location		
Left	44	39.3%
Right	68	60.7%
Smoke		
Yes	69	61.6%
No	43	38.4%
CCRT		
Yes	52	46.4%
No	60	53.6%
Cycle of chemotherapy before radiotherapy		
1–2	54	48.2%
Other	58	51.8%
Total cycle of chemotherapy		
4–6	69	61.6%
Other	43	38.4%
RT dose (Gy)		
<60	72	64.3%
≥60	40	35.7%
Radiotherapy time (day)		
<26	9	8.0%
≥26	103	92.0%
Targeted therapy		
Yes	17	15.2%
No	95	84.8%
PNI		
<45.15	18	16.1%
≥45.15	94	83.9%
PAR		
<4.03	21	18.8%
≥4.03	91	81.3%
PLR		
<162.81	88	78.6%
≥162.81	24	21.4%
NLR		
<2.2	73	65.2%
≥2.2	39	34.8%
LLR		
<4.32	89	79.5%
≥4.32	23	20.5%
dNLR		
<1.69	34	30.4%
≥1.69	78	69.6%
SII		
<815.1	93	83.0%
≥815.1	19	17.0%
SIRI		
<0.27	24	21.4%
≥0.27	88	78.6%
Brain metastases		
Yes	28	25.0%
No	84	75.0%

Abbreviations: CCRT: concurrent chemoradiotherapy; RT: radiotherapy; PNI: prognostic-nutrition index; PAR: platelet-albumin ratio; PLR: platelet-lymphocyte ratio; NLR: neutrophil-lymphocyte ratio; LLR: leukocyte-lymphocyte ratio; dNLR: derived neutrophil-lymphocyte ratio; SII: systemic immune-inflammation index; SIRI: systemic inflammation response index.

### Univariate and multivariate analysis of BM in LS-SCLC

Univariate and multivariate logic regression analysis were used to identify independent predictors of BM of LS-SCLC. Univariate regression analysis showed that the CCRT (*p* = 0.011; odds ratio (OR), 0.289; 95% confidence interval (CI), 0.111–0.752), RT dose (*p* = 0.008; OR, 0.300; 95% CI, 0.124–0.727), PNI (*p* = 0.002; OR, 0.189; 95% CI, 0.065–0.548), LLR (*p* = 0.025; OR, 3.034; 95% CI, 1.147–8.030), and dNLR (*p* = 0.011; OR, 0.313; 95% CI, 0.128–0.765) were the significant factors for a higher incidence of BM. Multivariate regression analysis further included the factors of a *p* < 0.05 in univariate regression analysis. Multivariate analysis showed that CCRT (*p* = 0.017; OR, 0.272; 95% CI, 0.093–0.795), RT dose (*p* = 0.011; OR, 0.262; 95% CI, 0.093–0.739), and PNI (*p* = 0.009; OR, 0.186; 95% CI, 0.052–0.657) were independent risk factors of BM and were included in the nomogram model. Table 2 revealed the results of univariate and multivariate analysis to identify the risk factors of BM for LS-SCLC.

**Table 2:** Univariable and multivariable logistic regression for analyzing the risk factors for developing BM in LS-SCLC patients.

**Clinicopathologic parameters**	**Univariate analysis**			**Multivariate analysis**		
	**OR**	**95% CI**	***p***	**OR**	**95% CI**	***p***
Age (years)						
≥66 vs. <66	0.510	0.137–1.901	0.316			
Tumor location						
Left vs. Right	0.663	0.269–1.638	0.373			
Smoke						
Yes vs. No	1.165	0.479–2.832	0.737			
CCRT						
Yes vs. No	0.289	0.111–0.752	0.011	0.272	0.093–0.795	0.017
Cycle of chemotherapy before radiotherapy						
1–2 vs. other	0.750	0.317–1.776	0.513			
Total cycle of chemotherapy						
4–6 vs. other	0.527	0.222–1.254	0.148			
RT dose (Gy)						
≥60 vs. <60	0.300	0.124–0.727	0.008	0.262	0.093–0.739	0.011
Radiotherapy time (day)						
<26 vs. ≥26	0.352	0.042–2.945	0.335			
Targeted therapy						
Yes vs. No	0.910	0.271–3.060	0.879			
PNI						
≥45.15 vs. <45.15	0.189	0.065–0.548	0.002	0.186	0.052–0.657	0.009
PAR						
≥4.03 vs. <4.03	0.600	0.214–1.681	0.331			
PLR						
≥162.81 vs. <162.81	2.179	0.826–5.748	0.116			
NLR						
≥2.2 vs. <2.2	2.360	0.983–5.668	0.055			
LLR						
<4.32 vs. ≥4.32	3.034	1.147–8.030	0.025	0.640	0.112–3.653	0.615
dNLR						
<1.69 vs. ≥1.69	0.313	0.128–0.765	0.011	0.358	0.075–1.717	0.199
SII						
<815.1 vs. ≥815.1	0.377	0.134–1.062	0.065			
SIRI						
<0.27 vs. ≥0.27	1.700	0.635–4.549	0.291			

Abbreviations: BM: brain metastases; LS-SCLC: limited-stage small cell lung cancer; OR: odds ratio; CI: confidence interval; CCRT: concurrent chemoradiotherapy; RT: radiotherapy; PNI: prognostic-nutrition index; PAR: platelet-albumin ratio; PLR: platelet-lymphocyte ratio; NLR: neutrophil-lymphocyte ratio; LLR: leukocyte-lymphocyte ratio; dNLR: derived neutrophil-lymphocyte ratio; SII: systemic immune-inflammation index; SIRI: systemic inflammation response index.

### Establishment and verification of predictive nomogram model

**Figure 1:**
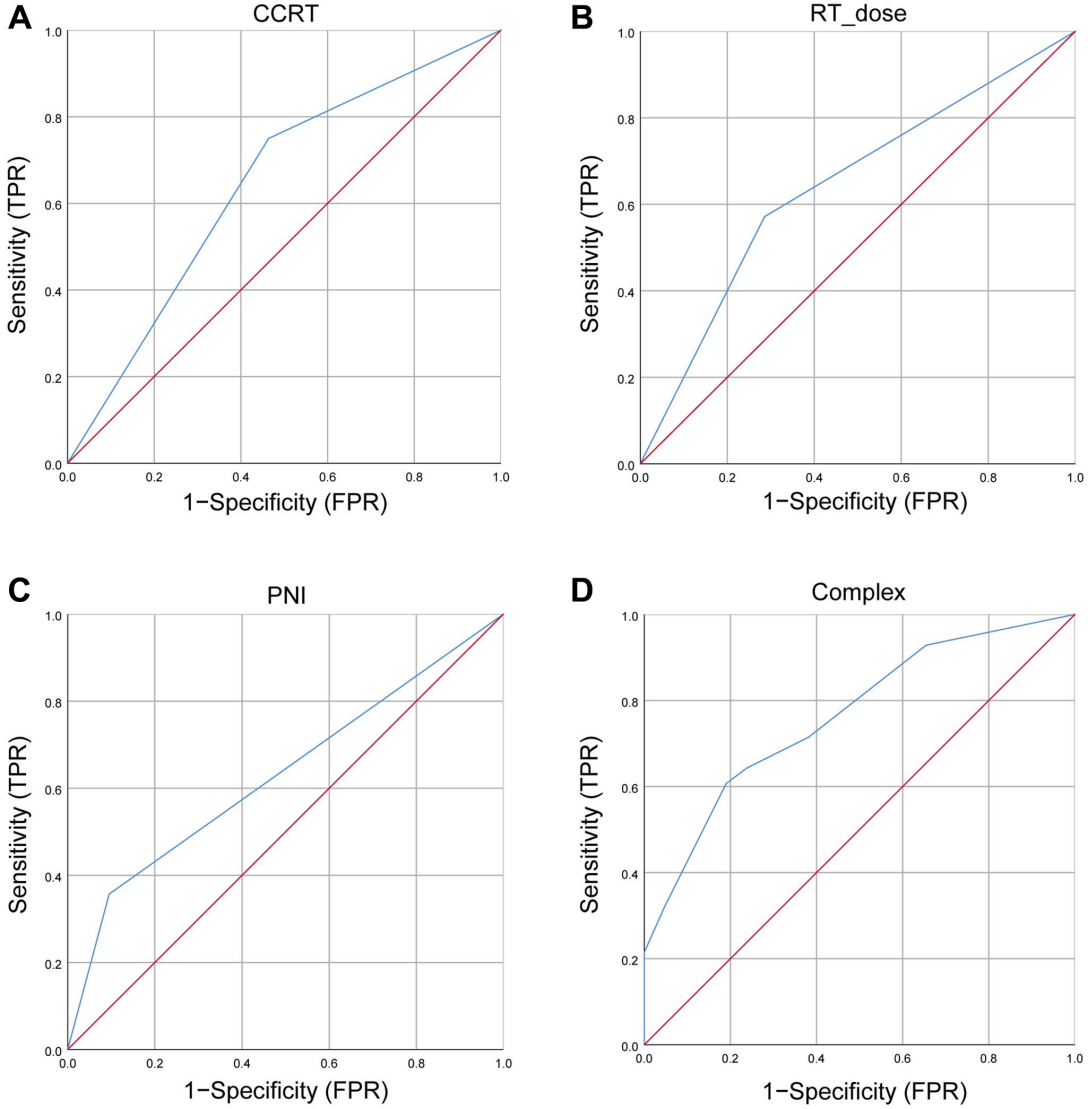
**The ROC curves for BM.** (**A**) The ROC curves of CCRT; (**B**) The ROC curves of RT dose; (**C**) The ROC curves of PNI; (**D**) The ROC curves of complex (CCRT, RT dose, and PNI). Abbreviations: ROC: receiver operating characteristic; BM: brain metastases; CCRT: concurrent chemoradiotherapy; RT: radiotherapy; PNI: prognostic-nutrition index; FPR: false positive rate; TPR: true positive rate.

**Figure 2:**
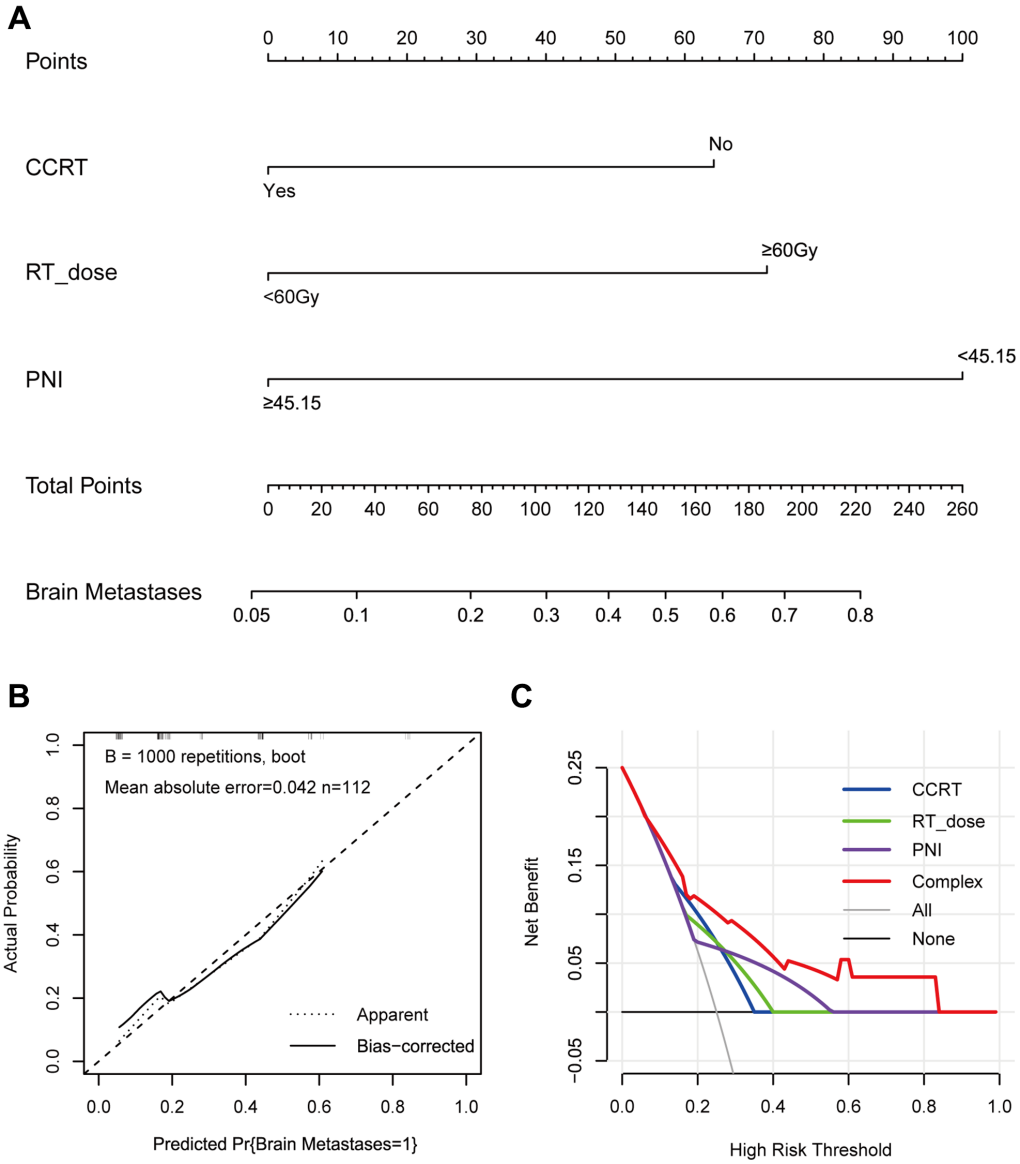
**The nomogram, calibration curve, and decision curve for predicting the probability of BM for the whole study population.** (**A**) A nomogram that integrates CCRT, RT dose, and PNI in LS-SCLC patients; (**B**) The calibration curve of the nomogram predicting the incidence of BM; (**C**) The decision curves of the nomogram predicting the incidence of BM. Abbreviations: BM: brain metastases; CCRT: concurrent chemoradiotherapy; RT: radiotherapy; PNI: prognostic-nutrition index; LS-SCLC: limited-stage small cell lung cancer.

To further identify the predicted values of risk factors in the multivariable logistic regression model, we developed nomograms to predict the BM of LS-SCLC patients. The ROC curves of CCRT, RT dose, PNI, and the complex (CCRT, RT dose, and PNI) were shown in Figure 1. The ROC curves revealed the area under the ROC (AUC) of the model was 0.764 (95% CI, 0.658–0.869), which was much higher than individual variable alone (CCRT: 0.643, 95% CI: 0.528–0.758; RT dose: 0.643, 95% CI: 0.521–0.764; PNI: 0.631, 95% CI: 0.502–0.759). The prediction nomogram model for BM was conducted and shown in Figure 2A. An individual’s final score is determined by adding up the points from each factor in the nomogram based on the relative importance of those factors. The calibration curve revealed favorable consistency between the observed probability and predicted probability for brain metastases for LS-SCLC patients (Figure 2B). The C-index for the prediction nomogram was 0.764 (95% CI: 0.658–0.869), which suggested the model’s good discrimination. In addition, the DCA was generated to determine the clinical benefit and practicality of this nomogram. As a result, the DCA demonstrated that the nomogram provides a satisfactory positive net benefit across the majority of threshold probabilities (Figure 2C). The DCA indicated that LS-SCLC patients could benefit from using the nomogram to predict BM probability.

### Correlation between nutritional index and inflammatory index

**Figure 3:**
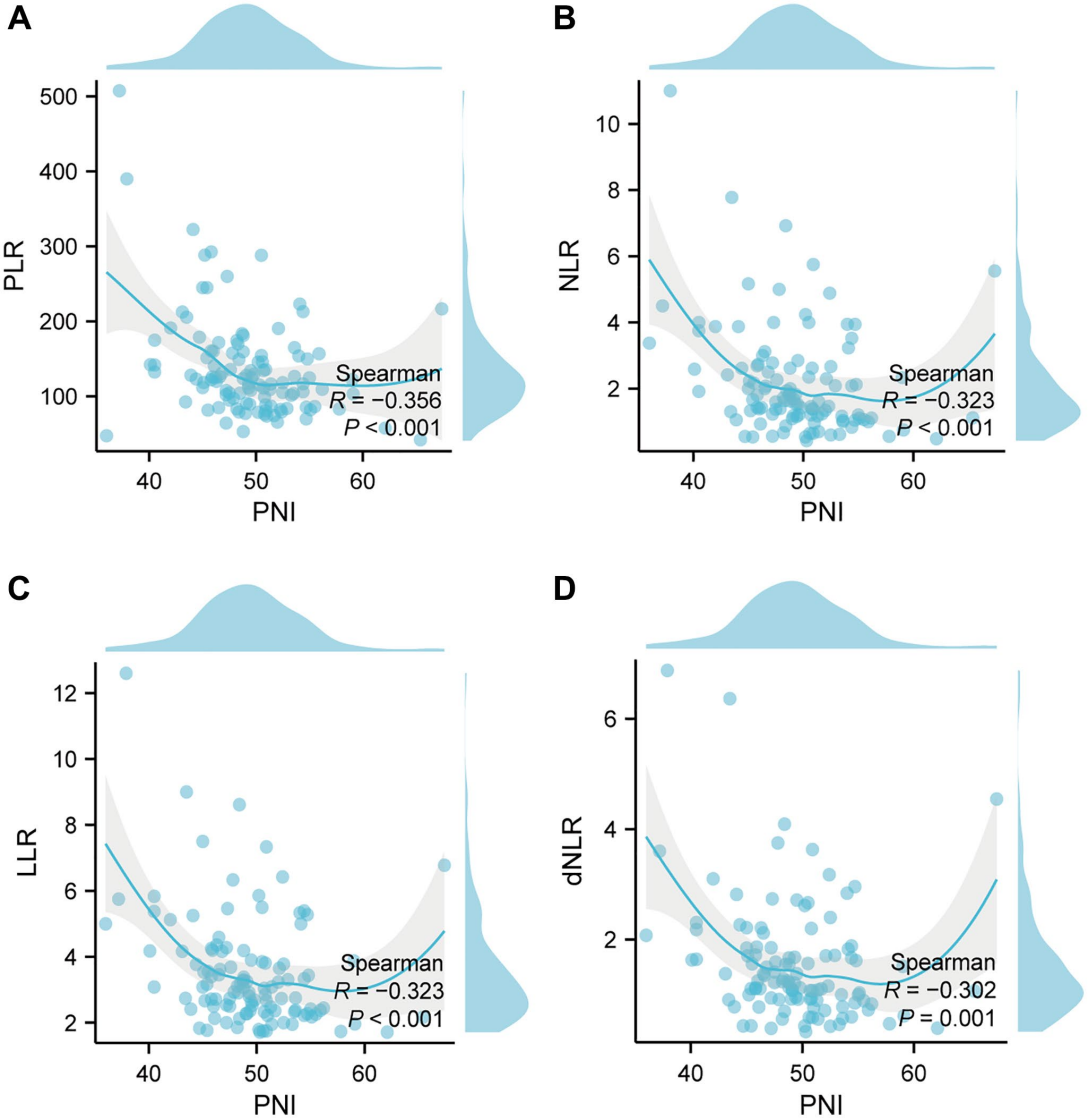
**Correlation between nutritional index and inflammatory index in the whole study population.** (**A**) The correlation between PNI and PLR; (**B**) The correlation between PNI and NLR; (**C**) The correlation between PNI and LLR; (**D**) The correlation between PNI and dNLR. Abbreviations: PNI: prognostic-nutrition index; PLR: platelet-lymphocyte ratio; NLR: neutrophil-lymphocyte ratio; LLR: leukocyte-lymphocyte ratio; dNLR: derived neutrophil-lymphocyte ratio; R: correlation.

We next performed Spearman correlation to explore the correlation between PNI, PLR, NLR, LLR, and dNLR. Spearman’s analyses revealed a negative correlation between PNI and PLR (R = −0.356, *p* < 0.001), PNI and NLR (R = −0.323, *p* < 0.001), PNI and LLR (R = −0.323, *p* < 0.001), PNI and dNLR (R = −0.302, *p* < 0.001) (Figure 3).

## DISCUSSION

SCLC is a highly aggressive malignancy, poorly differentiated tumor that develops from bronchial neuroendocrine cells, with a 5-year overall survival rate of 6–7% [14]. It is a well-known fact that at an early stage, this tumor will exhibit hematogenous metastases and lymph node metastases. Nevertheless, early-stage SCLC isn’t a common clinical scenario. SCLC poses a serious threat to the lives of patients [15]. Chemoradiotherapy (CRT) is the treatment of first choice for patients with stage III LS-SCLC. Despite CRT, a proportion of patients will develop BM during the follow-up period. The central nervous system is a known refuge for SCLC, which makes it difficult to implement effective measures against the disease. BM is a common cause of death in SCLC patients. The prognosis for patients with SCLC who present with BM is generally unsatisfied [16]. These patients place a heavy burden on the nursing staff, the society, and the family members who are caring for them. The importance of BM for SCLC patients cannot be overstated. It is only through early diagnosis and prompt treatment that their chances of survival can be improved [17]. However, due to individual differences, the risk of BM is inconsistent and unclear even in male SCLC patients at the same tumor node metastasis (TNM) stage. Therefore, it is crucial to find an efficient tool that predicts the risk and probability of BM in SCLC patients with the same gender and TNM stage.

Recently, the nomogram has been widely used in cancer research and has been validated as a trustworthy instrument for evaluating the risk events in cancer patients, which may help with developing treatment strategy and conducting clinical trials. Therefore, well-established predictive models can help to better stratify treatment and risk assessment of LS-SCLC patients. In this retrospective study, we analyzed clinical data from male SCLC patients with stage III from 2008 to 2020. Our aim was to determine the independent risk factors affecting BM in LS-SCLC patients and to construct and verify a nomogram that could predict BM. To the best of our knowledge, there is currently no relevant prediction model has particularly been constructed to predict BM in male patients with stage III LS-SCLC. It represents the first attempt to develop prediction nomogram for this specific population segment.

The current study introduced a nomogram for predicting BM in LS-SCLC patients after CRT. We analyzed the impact of age, tumor location, smoke, CCRT, cycle of chemotherapy before radiotherapy, total cycle of chemotherapy, RT dose, radiotherapy time, PNI, PAR, PLR, NLR, LLR, dNLR, SII, and SIRI on the risk of BM. Our results demonstrated that CCRT, RT dose, and PNI were the independent risk factors for BM development. Next, we constructed and verified the nomogram using the significant factors in the multivariate logic analysis to predict the incidence of BM in LS-SCLC patients. The calibration curve showed an excellent consistency and discriminative ability between predicted risk and observed outcome for the nomogram. In addition, the DCA also revealed the potential clinical benefit of the model for future. These findings lend credence to the idea that this nomogram could be used in clinical practice, particularly for the purpose of developing individualized treatment plans. The nomogram is a tool that can be used by physicians to perform metastatic screening on SCLC patients who have a high risk of developing metastasis in the brain. As a result, timely treatment strategies can be implemented for these patients.

Compared with CCRT, patients without CCRT are more likely to develop BM, which was in accordance with the previous researches [18, 19]. Topkan et al. showed a significant difference (*p* = 0.03) between the concurrent CRT group and sequential CRT group for the occurrence of brain metastases in lung cancer, demonstrating that concurrent CRT can reduce the risk of BM [18]. Similarly, Robnett et al. demonstrated that the timing of radiotherapy can affect the risk of central nervous system recurrence, with a 27% incidence of BM in those receiving induction chemotherapy before radiotherapy and 15% in those receiving concurrent CRT. The incidence of BM within 2 years was 39% and 20%, respectively [19]. Although acute toxic effects following sequential CRT are usually well tolerated by patients, but tumor resistance is a problem that cannot be overlooked. The optimal combination of chemotherapy and radiotherapy is still controversial, but some studies have reported that the 3-year survival rate of patients with LS-SCLC is better with concurrent CRT than with sequential CRT and consolidation CRT [20]. In addition, simultaneous chemotherapy and radiotherapy can enhance the killing of tumors and reduce the accelerated re-proliferation of tumor cells during the radiotherapy process. Chemotherapy and radiotherapy act on tumor cells of different phases separately, which not only complement each other in space and time, but also enhance the sensitivity of treatment and anti-tumor efficacy, which can achieve more effective clinical treatment effect [21]. Therefore, we can be certain that CCRT treatment is a more reasonable treatment plan for patients in good condition and that it is worthy of reference and application by the majority of clinicians.

As is known to all, radiotherapy is the crucial treatment for LS-SCLC patients. Nonetheless, whether RT dose are associated with BM remains controversial. In our study, RT dose more than 60 Gy was an independent risk factor for BM. Similar findings from previous studies were reported. Compared to receiving more than 60 Gy of radiation therapy, receiving less than 60 Gy of radiation therapy significantly improved 5-year overall survival (26% vs. 16%) and decreased the risk of recurrence (36% vs. 52%) [22]. Another study showed that disease progression rates of 73% and 76% were observed in patients who received less than 60 Gy versus those who received more than 60 Gy, respectively [23]. This international phase III study, RTOG-0538, also demonstrated no significant effect on survival at doses below 60 Gy versus those above 60 Gy [23]. This also breaks the long-held theory in radiation oncology that more treatment equals better outcomes. However, in clinical practice, the majority of patients with LS-SCLC still receive radiotherapy doses in excess of 60 Gy [24]. Accordingly, patients with LS-SCLC do not necessarily benefit more from longer radiotherapy courses. In addition, the underlying mechanism behind the correlation between RT dose and BM development needs further elucidation. One possible explanation is that high radiotherapy doses cause more damage to normal tissues that comprise the blood-spinal cord barrier, causing tumor cells to more readily infiltrate the damaged blood-spinal cord barrier, which in turn produces BM through the cerebrospinal fluid circulation.

The present study also identified PNI was significantly associated with BM development in LS-SCLC patients. Our study results revealed that the optimum cutoff values for PNI was 45.15. There is a lack of consensus regarding the optimal PNI cutoff value, despite the fact that certain pieces of research have revealed that PNI may be a predictive factor of lung cancer. Recent studies have indicated that the cutoff value for PNI is 47.23–52.525 [25–27]. Due to the heterogeneity between the investigations, the cutoff values were distinct. The variation in sample size is also a significant contributor to the inconsistent research findings. PNI is an important nutritional index composed of albumin and lymphocyte. Although the relationship between PNI and BM in LS-SCLC has not been reported, a large number of studies have found that malnutrition is strongly associated with tumor metastasis and survival outcomes. Studies have revealed a high prevalence of malnutrition among cancer patients, including lung cancer patients [28]. Malnutrition interacts with tumor invasion and metastasis. Tumor metastasis is an independent factor leading to malnutrition, while the formation of tumor metastases contributes to malnutrition in patients [28]. According to the current study, the specific mechanism may be related to impaired immune function, inflammatory response, leptin level, and autophagy of tumor cells in cancer patients [29–31]. Therefore, nutritional therapy is important for SCLC patients. There is no evidence that nutritional support promotes tumor growth, and an increasing number of clinical studies have shown that nutritional therapy not only meets the nutritional needs of patients, but also enhances their immunity, improves their tolerance to radiotherapy and chemotherapy, kills tumor cells directly or indirectly, and reduces the risk of recurrence. In conclusion, timely interventions should be made to improve the nutritional status of patients with malnutrition.

Despite the fact that CRT is initially effective in treating SCLC, the central nervous system is a common location for recurrence. Our study revealed the incidence of BM in initial male SCLC patients with stage III is 25%, which was consistent with previously reported researches. In one study, the probability of BM in SCLC patients with stage I, II and III was 7% (2/30), 25% (3/12) and 27% (7/26), respectively [32]. Another study showed that the rates of BM in SCLC patients with TNM pathologic stages I, II, and III disease were 6.25% (2/32), 28.2% (11/39), and 29.1% (16/55), respectively [33]. Similar results have been reported in other studies, where BM was found in <10% of SCLC patients with pathological stage I compared to >20% of those with pathological stage II or stage III [34–36]. In patients with LS-SCLC, prophylactic cranial irradiation (PCI) can reduce the occurrence of BM and improve survival outcome [37]. However, some patients still experience BM even after implementing PCI [38]. Besides, PCI-induced neurocognitive dysfunction is a clinical issue that cannot be neglected [39]. PCI was found to be beneficial for patients at high risk of BM but not for those at low risk of BM [40]. Therefore, not all LS-SCLC patients develop BM. We need to develop a reliable predictive model to accurately evaluate the risk of BM occurrence so that patients at low BM risk do not have to undergo PCI and thus avoid neurotoxicity. The aim of this study was to construct a model for BM prediction in stage III LS-SCLC patients, and individualized treatment based on BM risk could benefit more patients. This assists in advising SCLC patients on treatment-related decisions as a way to enhance or diminish the intensity of treatment.

Although there are several studies to construct nomograms in lung cancer patients diagnosed with BM. However, it is still unclear how to distinguish between patients’ risks of developing BM in LS-SCLC. Qiu et al. developed a nomogram for predicting brain metastasis free survival in SCLC patients [27]. This study only analyzed the prognosis and did not analyze the risk factors of BM of SCLC. In a different piece of research, the researchers also focused solely on the prognostic factors of BM in SCLC, despite the fact that the sample size was quite large [41]. Although the risk factors and prognosis factors of BM were evaluated simultaneously in another study, the number of variables included was few and the staging of patients was complicated [42]. As we know, LS-SCLC patients typically present in stage III. Since most SCLC patients are male and at stage III, we present the first study to examine the risk factors for BM in male SCLC patients with stage III. There is no relevant prediction model has particularly been constructed to predict BM for this subgroup population till date. It represents the first attempt to develop prediction model for this specific population segment. The purpose of this research was to develop a predicted model for brain metastasis in male SCLC patients with stage III and helped in the early identification of high-risk patients and the selection of individualized therapies.

However, some limitations must be acknowledged in the present study. First of all, this study is a non-randomized and retrospective study, which may result in inevitable selection bias. Second, due to the rarity of SCLC and the strict inclusion criteria, the number of patients included in this study was relatively small. Therefore, more cases need to be collected in future studies. Third, several potential risk factors for BM in SCLC, including tumor markers and lactic dehydrogenase, were not included in this study due to a lack of data. In addition, the model constructed in this study is only applicable to male SCLC patients with stage III and has no reference value for predicting BM in other SCLC patients. In this study, the vast majority of our patients were not receiving immunotherapy. This prevents us from evaluating the effect of immunotherapy in brain metastases. Finally, these findings should be validated by prospective randomized controlled studies with larger data cohorts.

## CONCLUSION

In general, the study revealed that CCRT, RT dose, and PNI were associated with BM occurrence in male SCLC patients with stage III. We established and verified a nomogram model that combines these variables to predict the incidence of BM in LS-SCLC patients. The nomogram had good performance for predicting the occurrence of BM, which aids in the early identification of high-risk SCLC patients and the selection of individualized therapies. Since the model has high reliability and clinical applicability, it can provide clinicians with theoretical guidance and treatment strategy making. Further multi-center large-scale prospective studies are necessary to validate the validity of our findings.

## MATERIALS AND METHODS

### Patient selection

In this retrospective study, patients who were first diagnosed with stage III SCLC from 2008 to 2020 in the Fujian Provincial Cancer Hospital were included. The inclusion criteria were as follows: (1) Patients with first diagnosis of SCLC that was confirmed by cytology or pathology; (2) Male patient; (3) Age ≥ 18 years; (4) Karnofsky Performance Status (KPS) ≥ 70 points; (5) Patients received combined chemotherapy and radiotherapy (RT). The exclusion criteria were as follows: (1) Underwent surgery; (2) Patients with incomplete clinical and laboratory data; (3) Patients with BM at first diagnosis; (4) incomplete therapeutic information; (5) Patients with a history of other malignancy. The laboratory test results were collected within one week before treatment. In this study, the clinical stage was defined by the 8th American Joint Committee on Cancer (AJCC) staging system for SCLC. Following data filtering, one hundred and twelve patients who fulfilled the above criteria were included in this analysis. The Ethics Committee of Fujian Provincial Cancer Hospital reviewed and approved this study.

### Treatment schedules

All patients received combined chemotherapy and radiotherapy. Etoposide, paclitaxel, or irinotecan was used in the chemotherapy regimens, and these drugs were combined with cisplatin, carboplatin, nedaplatin, or lobaplatin. The chemotherapy schedules adhered to the guidelines provided by the National Comprehensive Cancer Network (NCCN). All of the patients were scheduled to receive either three-dimensional conformal radiation therapy (3D-CRT) or intensity-modulated radiation therapy (IMRT). A 6MV medical linear accelerator was used for radiotherapy. Based on the guidelines of the NCCN, 45 Gy in 3 weeks (1.5 Gy twice daily [BID]) is superior (category 1) to 45 Gy in 5 weeks (1.8 Gy daily). When BID fractionation is used, there should be at least a 6-hour interfraction interval to allow for repair of normal tissue. If using once-daily RT, higher doses of 60–70 Gy should be used. The patients were received with a total dose of 42–69 Gy. In this study, concurrent radiotherapy within six cycles after the initiation of chemotherapy was defined as concurrent chemoradiotherapy.

### Definition of nutritional and inflammatory index

The prognostic-nutrition index (PNI), platelet-albumin ratio (PAR), platelet-lymphocyte ratio (PLR), neutrophil-lymphocyte ratio (NLR), leukocyte-lymphocyte ratio (LLR), derived neutrophil-lymphocyte ratio (dNLR), systemic immune-inflammation index (SII), and systemic inflammation response index (SIRI) were calculated as follows: PNI = the serum albumin level + 5 × the absolute lymphocyte count; PAR = the absolute platelet count/the serum albumin level; PLR = the absolute platelet count/the absolute lymphocyte count; NLR = the absolute neutrophil count/the absolute lymphocyte count; LLR = the absolute leukocyte count/the absolute lymphocyte count; dNLR = the absolute neutrophil count/(the absolute leukocyte count-the absolute neutrophil count); SII = the absolute neutrophil count × the absolute platelet count/the absolute lymphocyte count; SIRI = the absolute neutrophil count × the absolute monocyte count/the absolute lymphocyte count.

### Endpoints and follow-up

The endpoint is the development of BM in the SCLC patient. From the Electronic Medical Record System, we retrieved and reviewed the baseline clinical characteristics. Patients were followed up on at regular intervals (every 3–6 months) with a brain magnetic resonance imaging (MRI) after treatment. BM was diagnosed based on clinical manifestation and an enhanced MRI of the head. At the end of the follow-up period, all cases were censored.

### Statistical analysis

All data analysis used Statistical Product and Service Solutions (SPSS) version26.0 and R software version 4.0.2 (R Foundation). The optimal cut-off values of age, radiotherapy time, PNI, PAR, PLR, NLR, LLR, dNLR, SII, and SIRI were calculated by the Receiver Operating Curve (ROC). Specifically, univariate logic regression was applied to identify the potential risk factors of BM for further analysis in multivariate logic regression. Based on univariate analysis, we included the risk factors with *p* < 0.05 in the multivariate analysis to identify independent predictors of BM. A ROC and nomogram for predicting the incidence of BM were then conducted based on the independent risk factors calculated by the multivariate analysis. The calibration curves (internal validation was performed using 1000 bootstrap resamples of the training cohort) were generated to evaluate the consistency between observed probability and predicted probability of BM. The perfectly calibrated curve would exhibit with a 45 degrees curve. The decision curve analysis (DCA) was performed to assess the clinical benefit of prediction models. Finally, Spearman correlation was carried out to evaluate the correlations of nutritional index and inflammatory index. All tests were two-tailed and a *p*-value < 0.05 was considered statistically significant.

### Availability of data and materials

All relevant data are within the manuscript and its Supplementary Data Set.

## Supplementary Materials

Supplementary Data Set
